# Effect of interventions based on regular physical activity on weight management in adolescents: a systematic review and a meta-analysis

**DOI:** 10.1186/s13643-021-01602-y

**Published:** 2021-02-08

**Authors:** Babak Moeini, Forouzan Rezapur-Shahkolai, Saeed Bashirian, Amin Doosti-Irani, Maryam Afshari, Azam Geravandi

**Affiliations:** 1 Social Determinants of Health Research Center, Department of Public health, School of Public health, Hamadan University of Medical Sciences, Hamadan, Iran; 2 Department of Public health, School of Public health and Research Center for Health Sciences, Hamadan University of Medical Sciences, Hamadan, Iran; 3 Social Determinants of Health Research Center, Hamadan University of Medical Sciences, Hamadan, Iran; 4 Department of Epidemiology, School of Public Health and Research Center for Health Sciences, Hamadan University of Medical Sciences, Hamadan, Iran; 5 Department of Public Health, School of Public Health, Hamadan University of Medical Sciences, Hamadan, Iran

**Keywords:** Physical activity, Adolescent, Weight management, Meta-analysis

## Abstract

**Background:**

Physical inactivity is one of the major risk factors for non-communicable diseases. This systematic review and meta-analysis aimed to assess the effects of educational interventions on promoting regular physical activity in adolescent weight management programs.

**Methods:**

The relevant studies indexed in Embase, Web of Science, Scopus, and ProQuest databases were searched using keywords namely “Physical Activity, Adolescent, Weight Management, Body Mass Index (BMI)*,* Randomized Controlled Trials, and Clinical Trial.” Up to the end of March 2020, two authors independently screened the papers, extracted data, and assessed the methodological quality of the studies using Effective Public Health Practice Project (EPHPP) tool.

**Results:**

Out of 12,944 initial studies, 14 met the inclusion criteria after screening the titles, abstracts, and full texts of the papers. The participants in these studies were aged between 6 and 18 years, and 13 studies included participants from both sexes. Moreover, eight of them were performed as a controlled clinical trial. The overall estimate of the difference showed that the interventions improved weight loss which is a statistically significant finding. The participants in the intervention group had a weight loss of 1.02 kg compared to the control group at a 95% confidence interval (− 4.794–0.222).

**Conclusion:**

Published longitudinal data indicated that physical activity declines over the transition from adolescence to adulthood. Using the results of the study, policy-makers can design educational interventions using educational models and patterns.

**Systematic review registration:**

PROSPERO registration number: CRD42020173869

## Background

Despite scientific advances in healthcare, a worldwide increase in the number of chronic diseases due to mechanical life and poor eating habits is witnessed [1]. In recent decades, the epidemiological trend of diseases has changed so that infectious diseases have decreased and lifestyle-related diseases have increased [2].

According to available statistics, about 1.2 billion people in the world are overweighed [3]. Low physical activity is one of the major factors causing obesity. The amount of daily physical activity (PA) decreases with age [4–7]. A study by Cooper and Goodman analyzed pooled accelerometer data from more than 27,000 children and adolescents and showed that only 9% of the male and 2% of the female participants meet the World Health Organization recommendation of daily 60-min moderate-to-vigorous PA [8]. Over the past three decades, the prevalence of obesity and overweight in children and adolescents around the world has increased rapidly [9, 10]. According to the Centers for Disease Control and Prevention, the prevalence of obesity and overweight among adolescents (12–19 years old) has increased by 20.6% [11].

A sedentary lifestyle is the reason for many non-communicable diseases. Diseases such as primary hypertension, osteoporosis, and cardiovascular conditions, the effects of which appear at early and middle ages, are rooted in a sedentary lifestyle [12]. Regular physical activity, as an important health-promoting behavior, prevents or delays a variety of chronic illnesses and premature deaths, promotes mental health, reduces depressive and anxiety symptoms, and improves the quality of life [13–15]. Despite the benefits of physical activity, people in many countries, including Iran, do not engage in regular physical activity [16]. Hence, the World Health Organization (WHO) considers the level of physical activity (PA) as the first indicator of health in a society [17].

Currently, physical inactivity in young people is a big problem because the risk factors and unhealthy behaviors that contribute to heart disease in adulthood begin in childhood and adolescence [18]. Adolescence is the period of transition between childhood and adulthood and the level of physical activity decreases during this period [19]. Being a time-consuming and costly activity and fatigue from exercise prevent physical activity. Factors such as self-efficacy and support from others enhance physical activity in adolescents [20]. Adolescents, including students, are a justifiable group for assessing health-related behaviors because they constitute a large group in society [21]. A systematic review by Voskuil et al. [22] and a meta-analysis by Pearson et al. [23] assessed interventions to promote PA in adolescent girls in school and community settings.

Implementation of interventions is effective in improving the regular physical activity of adolescents and ensuring their health, as it can pave the way for the prevention of many diseases in adulthood. The main focus of these interventions is on weight management in adolescents through increasing regular physical activity. To the best of our knowledge, most weight-loss interventions and techniques are focused on the role of nutrition and obesity control in older people. There is a need for a systematic review of physical activity in adolescents. Studies have shown that interventions based on behavior change models and theory are more efficient to create a behavior change [24, 25]. Thus, the main objective of the current systematic review was to assess the effect of educational interventions on promoting regular physical activity in adolescent weight management programs.

## Materials and methods

This systematic review and meta-analysis is based on the PRISMA statement [26] and registered in PROSPERO (CRD42020173869).

### Search strategy

The search was done in PubMed, Scopus, Em base, Science Direct, Web of Science, and Pro Quest databases. Using the population model, the intervention, comparison, results, and design of the PICOS study were examined in papers published before the end of March 2020. The search strategy focused on three themes: participants (adolescents), outcomes (physical activity and weight management), and study type (Randomized Controlled Trials and experimental studies). The articles’ references were searched for reassurance. The Keywords derived from MeSH were “weight loss, weight management, obesity, adolescent, and randomized controlled trial.” The search strategy in the PubMed database was performed according to Appendix 1, and the necessary changes were made according to the necessity in each one of the databases.

### Article selection criteria

To study the entry and exit criteria of the study, the PICOS index (population under study, type of studies, types of interventions, and type of outcome) was used.

The population under study was the adolescent age group (between the ages of 11 and 19). The types of interventions included interventions conducted at individual, family, school, or hospital levels for adolescents. The types of studies were experimental and semi-experimental interventions and the subjects of studies were weight management interventions and increase of physical activity in different places. In addition, primary consequences in studies, i.e., increased physical activity, and secondary consequences, i.e., increased self-efficacy and change of attitudes and awareness, were examined. There was no publication date limitation and only studies published in English entered the study.

At first, all studies were reviewed by two researchers (AG and MA) based on the proposed search strategy, and all the obtained articles were entered into the EndNote software. Then duplicate items were removed in the EndNote. The title and summary of the relevant studies were independently examined by the two researchers in terms of relevance to the research question. The results obtained by them were matched and the differences were resolved by agreement or, if necessary, by a third researcher. To ensure that the study question was appropriate, the full text of the study would be reviewed and, if appropriate, the study question would be selected to assess the risk of bias in the next step.

### Statistical analysis

The statistical heterogeneity across the results of the included studies was explored using the Chi-squared test and quantified using *I*^2^ statistics. The *I*^2^ values of < 50%, 50–74%, and ≥ 75% were considered as low, moderate, or high heterogeneity respectively [29, 30]. Publication bias was evaluated visually by funnel plot; in addition, the Begg and Egger tests were used to examine statistical significance of this bias [27, 28].

### Summary measure

The mean difference of the effect of the intervention on the weight loss was calculated for each included study. The change score approach was used to obtain the effect size because the correlation between before and after measurements was more than 1/2. Meta-analysis was used to obtain the pooled effect size. The random-effects model was used to report the results of the meta-analysis [29] and the results were reported with a 95% confidence interval. Statistical analysis was performed using Stata 11 (StataCorp, College Station, TX, USA).

## Results

**Fig. 1:**
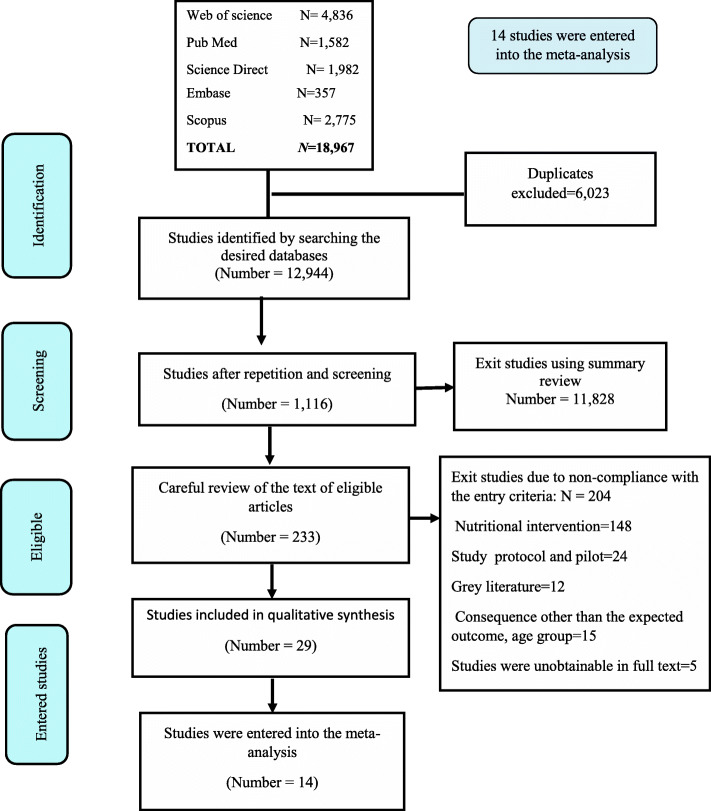
Flow diagram for identification, screening, eligibility, and inclusion of studies in systematic review and meta-analysis

The initial search yielded 12,944 abstracts, out of which, 11,828 articles were excluded. The remaining 1116 articles were reviewed and 233 were found eligible for the study. Then, the full texts were reviewed independently by the two researchers and the selection agreement between the reviewers was excellent Cohen’s kappa coefficient (0.739) [30]. The study selection process is illustrated in Fig. 1. Eventually, 14 studies were the meta-analysis phase [27–29, 31–41].

The strategies of the intervention in the studies were to hold training classes and film screening, counseling, practical training by a sports coach, motivational interviews, support from parents and peers, and school staff, application design, and use of Facebook groups.

The fourteen articles included eight RCT studies, one nRCT study, four CCT studies, and one experimental study. The total participants in these studies were 4437 individuals with age range between 6 and 18 years. The participants in thirteen studies were girls and boys [27–29, 32–38], and one study was performed only on girls [41] (Table 1).

**Table 1:** Summary of interventions to increase regular physical activity for weight management

No.	Author/Year/Location	Clinical trial design/randomization strategy	Participants	Intervention description	Percentage of response and duration of intervention and follow-up, theory and model used	Measurement of consequences	Results	Study quality
1	Simon 2008 France	Random cluster trial	C: *N* = 479; I: *N* = 475 Range 9. 9–13.8 years BMI: *I* = 21.32 (20.90; 21.75) kg/m^2^ C= 21.68 (21.45; 21.91) kg/m^2^ Target group=Student (six-graders)	In addition to the standard school curriculum, the intervention program includes - Three 50-min classes during the week - Holding sports and cycling competitions at school and encouraging and supporting parents and educators in improving the level of physical activity of adolescents through regular sessions.	Results were obtained from 848 students at the end of the second academic year. 778 in 3 years and 732 in 4 years The model was not used	The primary consequence is change in body mass index and the secondary consequence is the promotion of physical activity	Change in BMI and the level of physical activity	Strong
2	Hagstr¨omer 2009 Sweden	Randomization	*N* = 31 Exercisegroup (*n* = 16) Controlgroup∗ (*n* = 15) (Range 10–18 years) BMI: *I* = 31.9 (5.4); C = 33.4 (4.0) Target group = obese adolescent	Training sessions included: A variety of group activities over 13 weeks (1 h per week) conducted by management and coaching physiotherapist (one brisk walking session, 6 to 9 strength training sessions, and 10 to 13 swimming sessions).	The results were assessed in 16 adolescents in the intervention group and 15 adolescents in the control group The model was not used	The primary consequence is a change in body mass index and the secondary consequence is the promotion of physical activity	Increased physical activity *p* < 0.05	Weak
3	Ansari 2010 Egypt	Intervention in the target group of male and female students	*N* = 160 I: *N* = 80 ((35 boys, 45 girls) C: *N* = 80 ((35 boys, 45 girls) Mange age+15. 7 years BMI: *I* = 20.9 (4.1); C = 21.2 (3.6) Target group = adolescent school pupils	Students were offered two face-to-face sessions per week, each lasting about 30 min. There was one PE hour per week, which was much less than the international order for PA for teens, and attending these physical education classes were not mandatory, meaning students could not attend.	Data were collected at two time periods before and three months after the intervention. The model was not used	Primary outcome: increased physical activity secondary outcome change in the anthropometric index.	The results of a separate study of male and female students in which the weight decreased by 12.5% after three months in the intervention group and increased by 37.3% in the control group.	Strong
4	Lison 2010 Spanish	Three-arm interventions: two intervention groups and one control group	2 intervention groups I1: hospital, clinic group-based (GRX) *N* = 45 I2: home-based (HOX) *N* = 41 C: *N* = 24 BMI: HOX *I* = 29.7 3.7 GRX *I* = 28.5 3.8 C= 29.2 3.9 Target group = Overweight and obese children and adolescents,	The training was given to participants in the HOX group by a physical education instructor at the hospital. The program consisted of 5 sessions per week for 6 months (120 sessions) and the duration of each session was about 60 min. 5 min for warm-up and cooling (traction), 35 min of moderate aerobic activity, and 20 min of resistance training (low training exercises and high repetition).	The measurement was performed in two time periods before and 6 months after the intervention. The model was not used	Primary outcome: increase physical activity intervention and weight loss, and a change in body mass index in both groups	A significant decrease in body fat percentage and body mass index was observed in both intervention groups (4, 0.16), hospital-based group and; (4.4%, 0.23), located at home, *p* < .001	Weak
5	Maloney 2012 Massachusetts	Randomized controlled trial	Treatment group (*n* = 33) Comparison group (*n* = 31) Ages of 9 and 17 years with a body mass index (BMI) of 85–94% (overweight category). Range age = 9 and 17 years BMI (percentage): *I* = 96.57 (3.69) C = 96.64 (2.69) Target group = overweight and obese children and adolescents	Participants in the therapy group received a DDR X dance package. Each disc contained more than 100 songs of different styles. Participants were randomly assigned to the control group, followed by DDR software and dance packs for home use.	The measurement was performed two periods before and 12 weeks after the intervention. The model was not used	Preliminary results of the activity increase using accelerometer were evaluated	There was no significant change between pre- and post-intervention weight gain. 840. =p	Weak
6	II 2014 China	Nonrandom, controlled trial	*N* = 921 I: *N* = 388; C: *N* = 533 Aged 7 to 15 years BM *I* = 19.59 kg/m^2^ BMI: *I* = 19.12 (4.28) BMI: C = 19.93 (4.47) Target group = students (489 boys and 432 girls)	During 12 weeks, the intervention group participated in a multi-component physical activity intervention that included:-Education of physical activity-Programming for overweight/obese students, PA at home-Lecture for students and parents	The change in the studied variables (change in the level of physical activity and weight) occurred in two time periods before the intervention and 3 months after the intervention Models used: Ecological Social-ecological model	Changing the level of physical activity and BMI, HDL, LDL	The change in BMI in the intervention group (0.02–0.02 kg/m^2^) and change (MVPA) was significantly different from the control group (*p* = 0.001).	Strong
7	Simon 2014 France	Cluster-randomized controlled trial	*N* = 732 I: *N* = 275; C: *N* = 256 BMI= *I* = 18.6 (3.3) C = 18.8 (3.5) Target group= Students Adolescents	The standard the curriculum includes three sessions of 50 minutes of physical training per week plus intervention based on the dynamic interaction between PA factors and targeting individuals, family and their peers, and their living environment to promote the adoption of an active lifestyle at home.	Following a 30-month follow-up, body mass index, fat mass index, recreational PA (active movement at home/school/work, TV viewing time), attitude toward PA in adolescents were measured. The model was not used	Changes in indicators studied (BMI and fat)	The change was more than 1 week on TVT during the study with a change in additional BMI and FMI of more than 0.7 kg/m^2^. The introduction of TVT changes in the models reduced the effect of the intervention on additional BMI and FMI by 34.8% and 27.4%, respectively.	Medium
8	Galena 2014 France	Randomized-controlled study	*N* = 54 (28 in the SWLP group, 26 in the SWLP + MI group Ages of 11 and 18 years BM *I* = 29.57 kg/m^2^ SWLP + M *I* = 29. 56 ± 4.75 SWLP= 29. 59 ± 5.92 Target group= obese adolescents	Phase 1, making participant’s acquaintance and building awareness Phase 2, Alternatives and problem solving Phase 3, Goal setting and agenda-setting Phase 4, Behavior modification consequences and perspectives Behavior change feedback	Allocated to SWLP group (*n* = 34) Analyzed (*n* = 28) During 6 months Allocated to SWLP + MI (*n* = 28) Analyzed (*n* = 26)	Change in BMI Change in PA Change in motivational regulations Change in perceived competence	No differential change over time was found for the SWLP + MI group as compared to the SWLP group, suggesting a similar increase for both groups on this variable (*p* = . 34).	Strong
9	Direito 2015 New Zealand	3-arm parallel RCT	*N* = 51 (*I*: *N* = 16; C: *N* = 18) Range age = 14–17 BM *I* = 22.9 (SD 4.3) kg/m^2^ Zombies, Run = 23.17 (3.60) Get running = 21.85 (3.14) C = 23.43 (5.56) Target group=adolescents	Use commercially available applications. An 8-week training program	Measured in two stages before and 2 months later The model was not used	Primary outcome: cardiac and respiratory preparedness secondary outcomes: PA levels (acceleration and self-reporting), pleasure, psychological satisfaction, self-efficacy and acceptance, and program usability.	There was no intervention effect in the initial result using any of the programs. Compared to control, the fitness test time is − 28.4 seconds shorter and for the all-round program group and − 24.7 s for the unconventional program group.	Weak
10	Ruotsalainen 2015 Finland	Three-arm randomized controlled trial	(Fb + Act, 푛 = 15) (Feb, 푛 = 16) Control group (푛 = 15). BM *I* = 28.1 (SD 5.7) Fb + Act = 29.7 (8.1) Fb = 27.5 (4.2) C = 27.0 (3.8) Target group = obese adolescents	12 weeks of Facebook consultation and self-assessment of physical activity	Assess the level of physical activity before and 3 months after the intervention The model was not used	Physical activity and weight control	No intervention effect was observed in terms of changes in physical activity level or BMI from the beginning to 12 weeks after the intervention among the intervention and control group.	Medium
11	Chen 2016 Taiwan	Randomized controlled trial	*N* = 50 I: *N* = 25; C: *N* = 25 Age range of 12–15 years BMI= *I* = 27.90 2.71 C = 29.98 3.97 Target group= Obese young adolescents	The physical activity program included a variety of moderate-intensity exercises four times a week for 40 min (5 min to warm up and cool down, 30 minutes for the main workout) for 3 months. Each participant received a physical activity booklet with three sections: a warm-up, instruction manual, an exercise description, and a daily exercise newspaper.	The measurement was performed in three stages before the intervention, one week later and 3 months after the intervention The model was not used	Physical activity Body mass index and cardiovascular function	The physical activity program improved participants' fitness and obesity status.	Medium
12	HAM 2016 Butter	Experimental study	*N* = 75 I: *N* = 48; C: *N* = 27 Range age = 8–13. BMI= *I* = 24.35 (2.73) C = 24.22 (2.24) Target group = overweight/obese children	The TTM-based sports counseling intervention group consisted of 8 sessions over a 3-month period (4 consecutive weeks for the first month and 2 weeks for the next week). Each individual counseling session lasted 30 min.	TTM model for measuring in 2 time periods before the intervention and 6 months after the intervention was used.	Changes in physical activity were based on model structures (behavioral change, self-efficacy), blood sugar, and BMI.	Self-efficacy increased significantly in the experimental group. According to the change steps, 36.2% of the experimental group performed at least one stage of their exercise behavior compared to 17.4% of the control group.	Strong
13	Hollis 2016 Australia	School-based Randomized controlled trial	*N* = 1150 I; *N* = 425; C: *N* = 560 (Mean age =12 years) BMI= *I* = 19.90 (3.59) C = 20.19 (3.81) Target group = children and adolescents	The school intervention involves seven strategies for physical activity that include the following: Curriculum (strategy to maximize physical activity in physical education training, student physical activity programs, an improved school exercise program). 2. School environment (physical activity during school breaks, school policy change).	The measurements were made in the three-time periods. Before, 12 months, and 24 months after the intervention SDT and socio-ecological theory was used.	Changing physical activity Changing BMI	After the weight loss intervention and BMI, the students who were in the intervention group did not recover despite the increase in their physical activity.	Medium
14	Bagherniya 2018 Iran	Randomized controlled trial	*N* = 172 I: *N* = 87; C: *N* = 85 BMI= 29.47 (4.05) kg/m^2^ *I* = 29.2 (3.9 C = 27.2 (2.9) Target group = overweight and obese girl students	The activities of this trial included: sports, workshops, private counseling sessions in the field of physical activities, practical and competitive sports sessions, family sports sessions, text messages, and newspapers.	Measurements were performed in the time periods before, the first follow-up 3 to 5 months later, and the second follow-up 7 months after the intervention. SDT model was used	Changes in physical activity and BMI and model structures	PA and most of the psychological variables (self-efficacy, social support, and intention) increased significantly, the hours of watching television and computer games decreased significantly (*p* < 0.001).	Strong

A quality assessment was performed and four articles had poor quality [27, 28, 32, 36], four articles had medium quality [35, 37, 38, 40], and six articles had high quality [29, 31, 33, 34, 39, 41]. Cohen’s Kappa coefficient was used to evaluate the agreement between the two researchers when evaluating the quality of articles. The agreement between the two researchers was based on six components of the EPHPP tool. There was a good agreement between the researchers for attrition and a very good agreement was reached for the other components [42].

### Meta-analysis

The main indicator studied in this work was the effect of interventions and the results were calculated with a 1.02 (95% CI: 0.22, 4.79). A mean difference was used to combine the results of different studies. The overall estimate of the mean difference showed that the interventions had a statistically significant effect on weight loss.

**Fig. 2:**
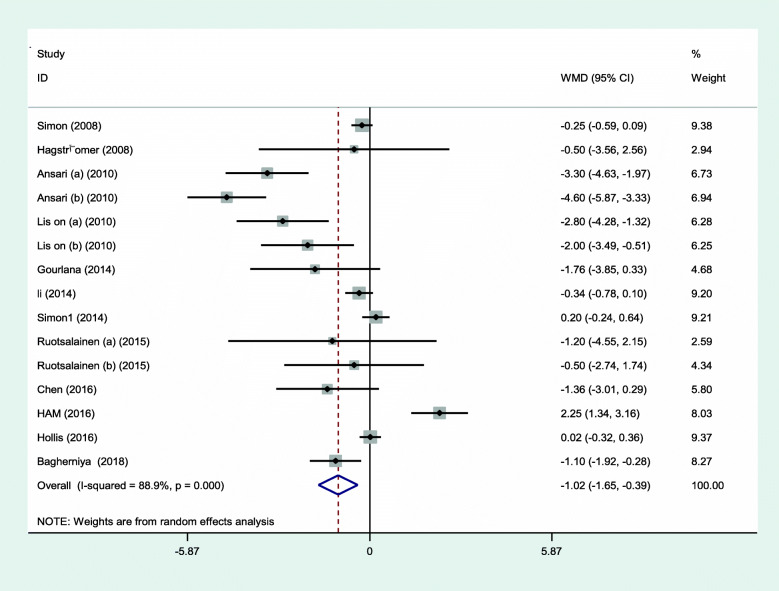
The effectiveness of interventions on physical activity and weight management in adolescents

**Fig. 3:**
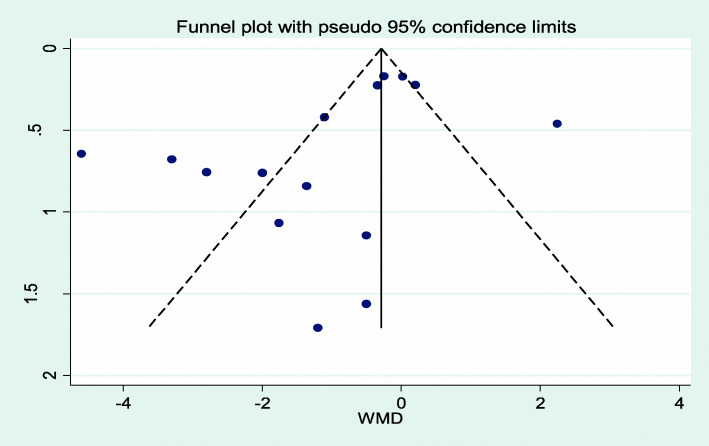
Funnel plot diagram shows bias in published studies

The overall estimate of the mean difference showed that the interventions had a statistically significant effect on weight loss. The subjects in the intervention group had a weight loss of 1.02 kg compared with the control group. The meta-analysis estimate was obtained with a CI of 95% (0.22–4.79). The statistical value of *I*^2^ in this analysis was 88.9%, which indicates the high heterogeneity of the study results (Figs. 2 and 3).

To minimize heterogeneity within the meta-analysis, studies were only included if they measured the same outcome measure (adjusted difference in mean values of PA/BMI in the intervention group compared with the control group at follow-up).

### Publication bias

According to the funnel plot chart, studies do not seem to be distributed symmetrically around its axis. However, the results of the Begg statistical tests (*p* = 0.656) and Egger (*p* = 0.071) indicate that there is no publication bias.

## Discussion

The objective of this systematic review was to investigate the impact and identify the types of interventions used to promote regular physical activity in adolescents’ weight management programs.

The interventions significantly increased the probability of regular physical activity and affected weight management and weight loss. Overweight and obesity are clear signs of an unhealthy lifestyle, a sedentary lifestyle, and an increased intake of high-calorie foods [43]. The trend of weight gain in children and adolescents (2–12 years) is more intensive than that of adults. Lifestyle changes from traditional to modern can lead to an increase in the prevalence of overweight and obesity [44].

Today, design and implementation of interventions based on health education theories are one of the most vital and specialized activities of health education and training professionals. A better understanding of behavior and the proper use of theories can enable us to use them intelligently and correctly [31].

Studies based on a theory of behavior change were significantly effective in increasing overall PA levels [45]. In the study of Rostami Moez et al., the intervention designed using a hybrid model was effective in preventing students from leaving physical activity programs [46].

Based on the classification of researchers, out of fourteen studies, five studies examined the effect of educational intervention on the level of regular physical activity and weight management in adolescents using health education and health promotion models [29, 34, 39–41].

The theories and models used in this study included the transnational model and social and ecological cognitive theory. It turned out that social cognitive theory was more common than other models so that it was the basis of action in three of the studies [34, 40, 41].

In addition to using theories and patterns, one of the most important topics in health education today is interventions in health promotion environments. The studies conducted in the systematic review were conducted in three different places, including schools, places of residence, and hospitals. Interventions to improve adolescent physical activity have shown that schools are better place to implement such interventions [47].

According to measurement indicators, physical activity increased in six studies; although, it was not possible to compare them due to the use of different measurement indicators and tools [28, 29, 31, 34–36]. The effectiveness of the intervention in short term (2 and 3 months) was examined in seven studies [28, 32–34, 36–38]. The duration of the study in four studies was 6 to 7 months [28, 34, 39, 41] and it was 12 to 48 months in the other three studies [31, 35, 40].

A review of physical activity promotion interventions from 2000 to 2011 showed that the different intervention approaches including awareness-raising and text messaging through mass media mobilization, mass support approach, and school-based interventions were effective in promoting physical activity [44]. The key strategies used in 10 studies [17–19, 23, 24, 28, 30–33] included practical classes of physical activity training, group support, counseling, and e-learning and physical activity behavior were the primary outcome. Weight loss was the secondary outcome and all of these factors can play an important role in promoting the physical activity behavior.

Evidences suggest that the use of multiple support strategies (parental and coaching support), sports coach, long-term intervention, and focusing the intervention solely on physical activity were important [48, 49]. In one study, the authors used a mobile application [36] and in another study, they used Facebook [37] to teach subjects.

The longest follow-up time period of studies reporting favorable intervention effects on PA was 2 weeks [50]. Analysis of groups by follow-up period showed that physical activity behavior was effective for 12–24 months. Therefore, it is recommended to follow this program uninterruptedly for a long period of time. The duration of the intervention in the studies was at least 2 months. The effectiveness in studies that used combined intervention for a longer period of time was higher than one-dimensional interventions [35, 40, 41]. Interventions that used different approaches were more useful than the interventions that focused only on the educational/behavioral aspect.

To plan effective intervention programs to increase physical activity and weight management, many researchers tried to identify the factors in performing physical activity and removing existing barriers. The most widely used model was the social cognitive model, which was used as a theoretical framework to increase the behavior of regular physical activity and weight management. Educations based on this model promote self-efficacy in overcoming barriers and increasing social support by friends and family [40, 41].

Therefore, it is possible to increase the level of physical activity in educational programs by increasing self-efficacy, skills training, and creating stronger social support [46].

There are many factors involved in failure to do regular physical activity. These barriers include time-consuming and costly nature, exercise-induced fatigue, and factors such as self-efficacy and support [20]. By providing appropriate education and covering these barriers, adolescents can be encouraged to engage in regular physical activity and weight management.

Many biases may affect the results of a meta-analysis and systematic study. In this study, there was no significant publication-bias. The discrepancies observed in the data due to the differences in the type of interventions and the implementation of interventions in different environments (school, home, hospital) as well as different countries affected the occurrence of a behavior. Studies in which the blindness measures had not been performed had a lower quality score.

There was a difference between the studies and one of the most important differences was the sample size. The difference in sample size could affect the results. The sample size of most of the studies was small [27, 28, 31, 33, 35–38] and the duration of follow-up interventions were mostly short. The results measured in the studies were mainly based on self-report. The intervention approach in educational studies affected the outcomes as well. Five studies used patterns and theories of behavior change. There were eight RCT studies [29, 31, 35–38, 40, 41], one nRCT study [34], four CCT studies [27, 28, 32, 33], and one experimental study [39]. Four articles had poor quality [27, 28, 32, 36], four articles had medium quality [35, 37, 38, 40], and six articles had a strong quality [29, 31, 33, 34, 39, 41]. The Kappa coefficient was used to evaluate the agreement between the two researchers as to the quality of articles. The agreement between the two researchers was based on six components of the EPHPP tool.

Quality assessment of studies based on criteria; Sample selection bias, study type, confounders, blinding, data collection method, and sample loss and exclusion were also studied. Also, studies were classified into weak, medium, and strong based on these criteria. If other criteria are selected, the quality assessment results may be different. No studies were excluded due to poor quality. One of the most important differences between the studies was the sample size, which affected the results. Also, in most studies, blindness was not performed, which was effective in their quality assessment score.

The reason for the high heterogeneity may be due to differences in the location of the studies, differences in their sample size, and differences in the quality of the studies.

The majority of the selected studies were conducted in high-income countries, indicating that there are fewer research works in low- and middle-income countries on this topic [50]. Moreover, in terms of the type of studies, out of eight articles, seven articles were based on RCT, which emphasizes the problems of conducting RCT studies in low- and middle-income countries. None of the studies examined the effect of economic condition on PA and weight management. It is necessary to study the relationship between socioeconomic status and its relationship to the level of regular physical activity especially in the developing countries.

Interventions based on psychological constructs, especially those based on a psychological theory, were more efficient. Moreover, the duration of follow-up interventions to examine the long-term consequences of studies, the use of approaches other than education, and the simultaneous use of legislative/technological approaches to achieve better results are important. Given that regular physical activity is determined by a wide range of psychological, social, and cultural variables. It is reasonable to consider these factors in interventions designed to improve physical activity in individuals, including self-efficacy, social support, and perceived benefits and barriers [51]. Training increased physical activity by increasing self-efficacy, skills training, and creating more social support.

One of the strengths of this study is the several electronic databases used to search the articles. As to the weaknesses, limitation of the search to electronic databases due to time constraints and failure to encompass the barriers to physical activity (psychological, supportive, information barriers) are notable.

### Study limitations

The results provided a valuable perspective; however, unavailability of the English text of some articles in databases and journals and the inadequate description of the implementation method were among the major limitations. Another limitation was that these studies did not show which component of the intervention was more efficient.

## Conclusion

Health education interventions and programs have a significant impact on regular physical activity. Future works need to address the methodological weaknesses of the current study. Physical activity should be evaluated objectively especially outside of school as diversity of the results affects the quality of studies. Future interventions should include the measurement of psychological structures, especially if they are based on a psychological theory or when psychological mediators are considered. Evidences about the effectiveness of interventions help us designing better interventions (production of appropriate audio-visual print materials in the field of advantages and disadvantages of regular physical activity, types of physical activity, the correct way of physical activity, and its transmission through appropriate channels). Longer follow-ups are needed to study the long-term consequences of interventions. Moreover, larger sample size, approaches other than education, and simultaneous use of legislative/technological approaches to achieve the desired results are recommended.

## Appendix

First line: search for the physical activity part

#1: Exercises [Mesh Term]

#2: " Physical Activity" [tw]

#3: Activities [tw]

#4: Activity [tw]

#5:"Physical Exercise" [tw]

#6:" Physical Exercises" [tw]

#7:"Acute Exercise"[tw]

Second line: search for weight management

#9: Weight Loss [Mesh Term]

#10: Weight Reduction Programs [[Mesh Term]

#11:" Weight management"[tw]

# 12: BMI [tw]

#13: fat [tw]

# 14: overweight [tw]

#15: obesity [tw]

Third line: Search for the adolescent par**t**

#17: Adolescent [Mesh Terms]

#18: Teen [tw]

#19: Adolescence [tw]

#20: Teenagers [tw]

#21: Youth [tw]

Line 4: Search for the study type section

#23: "experimental studies" [tw]

#24: Non-Randomized Controlled Trials as Topic [Mesh Term]

#25: Randomized Controlled Trials as Topic [Mesh Term]

#26: randomized clinical trial [tw]

#27: non-randomized clinical trial [tw]

#28: "follow up studies "[tw]

#29: "prospective studies"[tw]

#30: "crossover studies"[tw]

## Abbreviation

WHO: World Health Organization
PRISMA: Preferred Reporting Items for Systematic Reviews and Meta-Analysis
CCT: Controlled clinical trial
RCT: Randomized controlled trial
PICOS: Population, Intervention, Comparison, Outcome, Study type
EPHPP: Effective Public Health Practice Project
BMI: Effective Public Health Practice Project
NCCMT: National Center for Methods and Methods Cooperation
